# The Phylogeographic and Spatiotemporal Spread of HCV in Pakistani Population

**DOI:** 10.1371/journal.pone.0164265

**Published:** 2016-10-20

**Authors:** Noor-Ul-Huda Ghori, Atif Shafique, Muhammad Qasim Hayat, Sadia Anjum

**Affiliations:** 1 School of Earth and Environment, The University of Western Australia, Perth, Australia; 2 Atta-Ur-Rahman School of Applied Biosciences (ASAB), National University of Sciences and Technology (NUST), Islamabad, Pakistan; 3 Department of Biology, College of Sciences, University Of Hail, PO Box 2440, Hail, Kingdom of Saudi Arabia; Kaohsiung Medical University Chung Ho Memorial Hospital, TAIWAN

## Abstract

Hepatitis C Virus (HCV) is the most prevalent human pathogen in Pakistan and is the major cause of liver cirrhosis and hepatocellular carcinoma in infected patients. It has shifted from being hypo-endemic to being hyper-endemic. There was no information about the origin and evolution of the local variants. Here we use newly developed phyloinformatic methods of sequence analysis to conduct the first comprehensive investigation of the evolutionary and biogeographic history in unprecedented detail and breadth. Considering evolutionary rate and molecular-clock hypothesis in context, we reconstructed the spatiotemporal spread of HCV in the whole territory of its circulation using a combination of Bayesian MCMC methods utilizing all sequences available in GenBank. Comparative analysis were performed and were addressed. Whole genome and individual gene analysis have shown that sub-types 1a, 1b and 3a are recognized as epidemic strains and are distributed globally. Here we confirm that the origin of HCV 3a genotypes is in South Asia and HCV has evolved in the region to become stably adapted to the host environment.

## Introduction

Hepatitis C virus (HCV) is a pandemic human virus. It’s a leading cause of chronic liver diseases including hepatocellular carcinoma and liver cirrhosis. Worldwide about 200 million people are infected with the virus. Approximately, 3–4 million people are diagnosed with new infections annually [1]. In Pakistan, almost 10 million people i.e. 6% of its population are reported to be infected with the virus[2]. HCV is a blood-borne pathogen and the main cause of virus transmission is a blood transfusion. Other risk factors include medical and dental procedures with unsterilized equipment, hemodialysis, organ transplants from HCV-infected patients, needle injuries, piercing and tattooing in unhygienic circumstances and intravenous drug usage.

**Fig 1:**
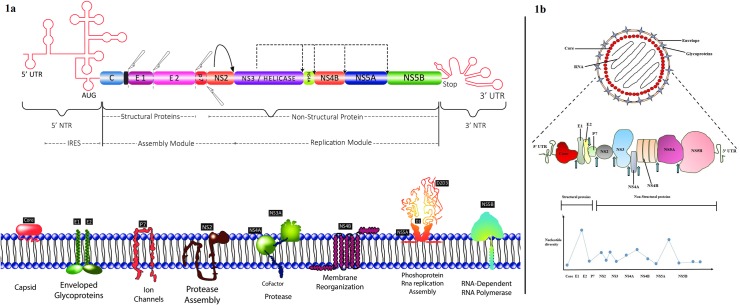
(a) and (b) shows the HCV structure and genome organization. The arrows shows the cleavage sites that enable protein maturation. (c) Shows nucleotide diversity graph of individual genes in HCV genome. Some of the genes are highly conserved whereas, others are highly variable having a hyper variable region.

HCV is a member of family *Flaviviridae* representative of genus *Hepacivirus*. The virus is enveloped and carry a ~9.6kb RNA single-stranded genome of positive polarity. It contains a single open reading frame (ORF) that encodes 11 proteins as shown in Fig 1A and Fig 1B.

HCV RNA replication is extremely efficient with a production rate of 10^12^ virions per day in a single individual [3] and a high error rate of approximately 1 in 100 000 bp copied [4, 5] which causes high genetic heterogeneity in the viral genome leading to 7 genotypes and more than 70 subtypes characterized so far [6]. These genotypes refer to the genetically distinct groups of HCV isolates which arise during evolution. At genotype level the divergence in the nucleotide sequence exhibited by the virus is 30% whereas at subtype level it’s 20% [7, 8]. One of the most important features of HCV genotype is its phylogenetic severalty, each genotype have close similarities to the individual branch of the phylogenetic tree of HCV. The subtypes are also important in view of epidemiology as they are reported to be associated with a pattern of spread or certain groups [9]. Interestingly, the seven genotypes not only diverge in a phylogenetic tree, they also vary in geographical distribution and their genetic diversity. HCV phylogeny resembles a shape of star with six arms [10, 11]. Similarly, the sub-types are significant in studying the effects, causes and patterns of the HCV [9].

Finally, HCV exists as closely related species known as quasispecies within an individual. Full length sequences show 90.8%- 99% similarity between quasispecies [12]. The occurrence of various genotypes correlates to their location. Genotype 1 and Genotype 2 are prevalent in West and Central Africa. In South Asia most of the infections are due to Genotype 3. In Africa and the Middle East, Genotype-4 is more common whereas, Genotype 5 and 6 have been found locally in South Africa and South-East Asia respectively [13, 14]. Asia and Africa have the highest prevalence rate in the world whereas Europe, Australia and North America are the lowest prevalence regions [15]. Sub-types 1a, 1b and 3a are recognized as epidemic strains and are distributed globally having low genetic diversity. Due to this scientist have concluded that the genetic diversity of endemic strains is greater than epidemic strains i.e. the low heterogeneity of epidemic strains make them highly stable. Various studies show that genotype 3 is most prevalent among the Pakistani population affecting about 78.96% of diagnosed patients. The most common subtype is 3a attributing 58.01% followed by genotype 3b [16].

Researchers have successfully revealed route of virus acquisition specific to a genotype or subtype [17–19]. A significant amount of data has been collected related to HCV genotypes. However, no comprehensive study regarding the origin and evolution of the local variants has been carried out till now. Keeping in mind the importance of geographical occurrence, clinical outcome and HCV strain relation, the current study has been designed. We have attempted to trace the origin, genetic diversity and molecular evolution of HCV infected population in Pakistan using coalescence theory and newly developed phyloinformatic methods. The coalescence theory’s hypothesis says that all individuals have a common ancestor and the population that has evolved independently tends to genetically diverge over time. Analysis of HCV sequences using this theory and molecular clock hypothesis enables us to predict HCV population dynamics since the time of its discovery which can be used to estimate viral epidemic behavior. The outcome of the study will help to develop better therapeutic interventions to overcome the disease.

## Materials & Method

### Data Collection

A total of 1,17,000 sequences (Partial/Complete genome) of HCV were retrieved from publically available database (GenBank) since inception to date into Geneious v 8.1.5 (http://www.geneious.com, [20]). These sequences belonged to different countries of the world and were renamed as:

Accession number: Country: Date: Subtype

Following inclusion criteria was fixed for sequences included in the study (a) sequences should be already published in peer reviewed journals; (b) there should be no ambiguity regarding the subtype consignment of each sequence; (c) all sequences should be non-recombinants; (d) country of the origin of sequence should be known and clearly established; (e) isolation date or publication date is clearly established in the original publication. Sequences with incomplete information were deleted.

### Study Population

For this study we collected HCV complete genome sequences of genotype 3a from Pakistan and worldwide and also sequences of HCV genotype 1a, 1b, 3a, 3b from Pakistan and worldwide. Whole genome sequences were edited manually to obtain individual genes of each genotype. The study was conducted using NS5B gene. Other genes were not considered for either being highly conserved or too variable that they could not be aligned unambiguously. As a large number of sequences were available and including all of them would have considerably increased computational burden, two representative sequences were selected from each country for each genotype. This was done by constructing Neighbor joining trees using Jukes and Cantor Model (JC; [21]) of individual genes.

### Evolutionary Model Selection

J-Model Test v2.1.6 [22, 23] was applied and simplest evolutionary model was selected that adequately fit the data matrix according to the Akaike Information Criterion (AIC). For complete genome sequences the best fit model is General Time Reversible GTR + I + G (012345).

### Phylogenetic Reconstructing Using Maximum Likelihood

Multiple alignments of NS5B region sequences were created and manually edited to form a data matrix. For complete genome two sets of nucleotide sequences were prepared for phylogenetic analysis; One set contained HCV genotype 3a consisting of 23 sequences and the other set contained all the genotypes and subtypes of HCV worldwide consisting of 102 sequences. Maximum likelihood phylogenetic trees were inferred using PhyML [23] program plugin available in Geneious v8.1.5 program (http://www.geneious.com, [20]). Boot strap analysis was performed using 1000 replicates.

### Bayesian Coalescent Analysis (Dated Phylogeny)

Bayesian coalescent analysis was performed using Bayesian Evolutionary Analysis Sampling Trees program (BEASTv1.8.2) [24]. Simplest evolutionary model was selected using J-Model Test v2.1.6 [22, 23]. Since we had no prior reason to assume a particular model of change in viral population size overtime, we used recently developed Bayesian Skyline plot to estimate epidemic history [25]. The sequences from Pakistani population in our database were sampled over a limited time frame and therefore, did not contain sufficient information about evolutionary rate. Therefore, we employed informative prior distributions for the NS5B evolutionary rates. We used 5.0×10–4 ± 1.7×10–5 substitutions/site/year for NS5B [26–28]. For complete genome analysis we used 1.289 × 10^−3^ ± 1.47 × 10^−5^ substitution per site per year evolutionary rate.

As recommended, un-correlated log-normal model for relaxed molecular clock was applied to all analysis. This model takes into account the changes in evolutionary rate among lineages [25]. MCMC chains were run for at least 50 million generations or till effective sampling size (ESS) value became >200 and sampled every 15000 steps [29]. For complete genome analysis MCMC run contained at least 140,798,000 states and sampled once every 10,000 steps. The posterior samples were analyzed using Tracer v1.5 program (available from http://beast.bio.ed.ac.uk/Tracer). Uncertainty in the estimates was indicated by 95% highest posterior density (95% HPD) intervals. Posterior distributions of the analysis were calculated after a burn-in of 10% using Tree annotator v1.5.2 (http://beast.bio.ed.ac.uk) and trees were visualized and formatted using FigTree (available from http://tree.bio.ed.ac.uk/software/figtree/).

### Phylogenetic Analysis Using Parsimony & Other Methods

PAUP (v. 4.0) a Geneious plugin was used to infer maximum likelihood (ML) phylogenetic trees [30]. These trees are generated with the best-fitting nucleotide substitution model General Time Reversibility (GTR) using estimated proportion of invariable sites. Best trees of HCV complete genome of genotype 3a were constructed from 10 iterations of the likelihood ratchets by using Heuristic maximum likelihood tree search parameter. Bootstrap value set for the analysis was 1,000. Best tree generated from each iteration was compared and consensus tree was performed by PAUP program.

## Results

### Phylogenetic Analysis of HCV Genotype 1a

**Fig 2:**
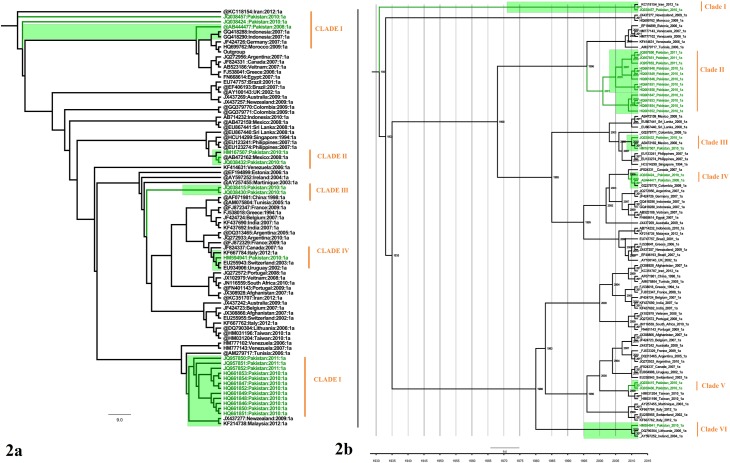
(a) Maximum likelihood phylogeny of NS5B 349bp long fragment gene reconstructed for Pakistani sequence origin of genotype 1a (Highlighted in green) using (GTR+I+G) model. All samples GenBank accession numbers, countries of origin and years of sampling are shown. Individual clades are marked on the right. (b) A time-scaled phylogenetic tree estimated using NS5B 349bp long fragment gene of genotype 1a. Each branch is led by an isolate and is denoted by the following format: GenBank Accession number: Country: Date: Subtype. The branch lengths represent the evolutionary time that is measured by the grids corresponding to the timescale (years) shown at the tree base. The node labels represent the time of divergence of the respective node. To simplify the tree, the estimated 95% HPD are shown against MRCA and Pakistani clades only.

The ML phylogenetic analysis of NS5B gene S1 Table showed that HCV genotypes 1a origin in Pakistan is polyphyletic and has occurred due to multiple outbreaks at different points in time. It is also observed that Pakistani sequences are more closely related to each other than to sequences from other countries. NS5B gene phylogeny is shown in Fig 2A. The phylogenetic tree clearly formed six clades. It is seen in clade II that Pakistani sequences recurrently clustered together. The Bayesian time scaled phylogenetic tree of NS5B gene agreed closely with the ML tree with minor exceptions. The estimated date for most recent common ancestor (MRCA) was found to be 1931 (95% HPD: 1382–173). The divergence date of individual clades can be seen on the tree in the Fig 2B.

### Phylogenetic Analysis of HCV Genotype 1b

**Fig 3:**
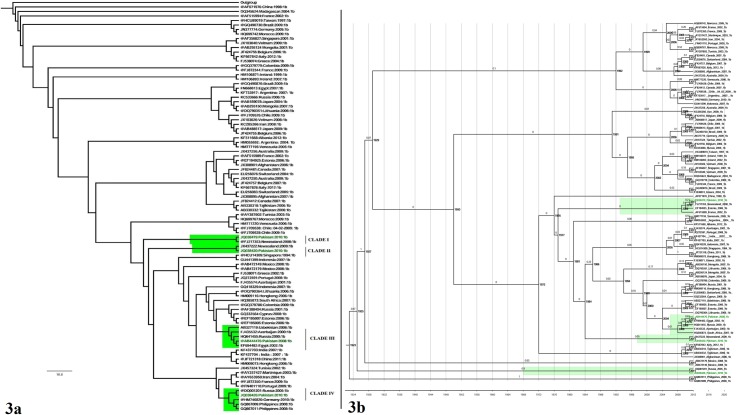
(a) Maximum likelihood phylogeny of NS5B 330bp long fragment gene reconstructed for Pakistani sequence origin of genotype 1b (Highlighted in green) using (GTR+I+G) model. All samples GenBank accession numbers, countries of origin and years of sampling are shown. Individual clades are marked on the right. (b) A time-scaled phylogenetic tree estimated using NS5B 330bp long fragment gene of genotype 1b. Each branch is led by an isolate and is denoted by the following format: GenBank Accession number: Country: Date: Subtype. The branch lengths represent the evolutionary time that is measured by the grids corresponding to the timescale (years) shown at the tree base. The node labels represent the time of divergence of the respective node. To simplify the tree, the estimated 95% HPD are shown against MRCA and Pakistani clades only.

ML and Bayesian dated phylogenetic analysis were performed on NS5B gene of genotype 1b (S1 Table). The resulting ML phylogeny is shown in Fig 3A. The phylogenetic tree consists of four clades containing Pakistani sequences. These sequences have clustered with sequences from New Zealand, Egypt and Russia. The tree showed a polyphyletic origin of HCV genotype 1b in Pakistan. The dated phylogeny were similar to ML trees Fig 3B. The divergence date of MRCA for was found to be 1923 (95% HPD 1347–283). Clade I and IV showed strong statistical support whereas, clades II and III were not well supported by posterior probability.

### Phylogenetic Analysis of HCV Genotype 3a

**Fig 4:**
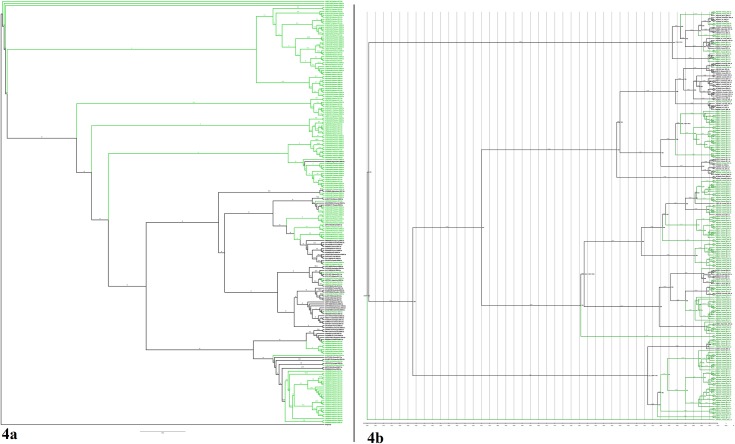
(a) Maximum likelihood phylogeny of NS5B 381bp long fragment gene reconstructed for Pakistani sequence origin of genotype 3a (Highlighted in green) using (GTR+I+G) model. All samples GenBank accession numbers, countries of origin and years of sampling are shown. Individual clades are marked on the right. (b) A time-scaled phylogenetic tree estimated using NS5B 381bp long fragment gene of genotype 3a. Each branch is led by an isolate and is denoted by the following format: GenBank Accession number: Country: Date: Subtype. The branch lengths represent the evolutionary time that is measured by the grids corresponding to the timescale (years) shown at the tree base. The node labels represent the time of divergence of the respective node. To simplify the tree, the estimated 95% HPD are shown against MRCA and Pakistani clades only.

ML and dated phylogeny of NS5B gene of genotype 3a S1 Table showed very similar results. The tree confirmed the polyphyletic origin of genotype 3a in the country Fig 4A. Pakistani isolated were found to be closely related to many countries including Portugal, Thailand, Vietnam, India, Argentina, UK, Brazil, Russia, Afghanistan and Mexico however, many of the clades were not well supported by bootstrap value. The ML and dated phylogeny of NS5B gene was very diverse and depicted the inter country dispersal and high prevalence of HCV genotype 3a over the years. The whole tree dated back to 1800. The divergence date of only the large clusters are shown in Fig 4B. The best probability support was seen in the clades clustering Pakistani sequences together confirming the inter country dispersal of the virus.

### Phylogenetic Analysis of HCV Complete Genome 3a

**Fig 5:**
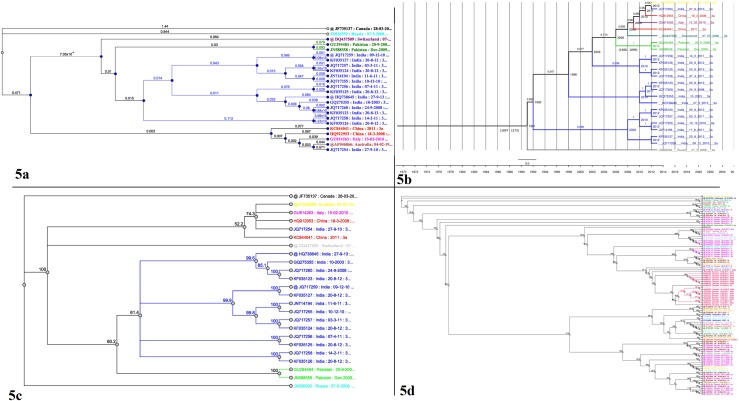
(a) Maximum likelihood phylogeny of HCV complete genome reconstructed for Pakistani sequence of genotype 3a. (GTR+I+G) model was used for analysis. The confidence support value of nodes was estimated using 1,000 bootstrap replicates. Pakistani sequences ae marked with green color. All samples GenBank accession numbers, countries of origin and years of sampling are shown. (b) A time-scaled phylogenetic tree estimated of genotype 3a of complete genome using Bayesian MCMC approach. Branch length represents the evolutionary time that is measured by the grids corresponding to the timescale (years) shown at the tree base. Estimated 95% HPD are shown against Pakistani clades only. Phylogeny branches and sequence names are colored according to the country. (c) Phylogenetic analysis using Parsimony and other methods of HCV complete genome reconstructed for Pakistani sequence of genotype 3a. (GTR+I+G) model was used for analysis. The confidence support value of nodes was estimated using 1,000 bootstrap replicates. Pakistani sequences ae marked with green color. All samples GenBank accession numbers, countries of origin and years of sampling are shown. (d) Maximum likelihood phylogeny of HCV complete genome reconstructed for all the genotypes (1, 2, 3, 4, 5 and 6). (GTR+I+G) model was used for analysis. The confidence support value of nodes was estimated using 1,000 bootstrap replicates. All samples GenBank accession numbers, countries of origin and years of sampling are shown.

The ML phylogenetic analysis of complete genome of HCV genotype 3a (S1 Table) showed that its origin in Pakistan is monophyletic. It has been observed that Pakistani sequences are more closely related to each other than to sequences from other countries. The gene phylogeny is shown in Fig 5A. Pakistani sequences form a single clade in the phylogenetic tree and share a closer common ancestor. The tree contains an outgroup sequence from Canada of subtype 4a. The Bayesian time scaled phylogenetic tree of complete genome agreed with the ML tree. The tree confirmed the monophyletic origin of genotype 3a in the country. The estimated date for MRCA was found to be 1992 (95% HPD: 1897, 1273). The divergence date of individual clades can be seen on the tree in Fig 5B. PAUP analysis of complete genome also showed that HCV genotype 3a origin in Pakistan is monophyletic. The gene phylogeny is shown in Fig 5C. Pakistani sequences share a closer common ancestor and forms a single clade in the phylogenetic tree.

The ML phylogenetic analysis of complete genome of genotype 1, 2, 3, 4, 5 and 6 (S1 Table) showed that genotype 3a origin in Pakistan is polyphyletic. Pakistani sequences forms two clades in the phylogenetic tree. The gene phylogeny is shown in Fig 5D. The tree contains an outgroup sequence from Rodent Hepacivirus (KC411777). The tree explains the evolution of HCV genome. Multiple clades are present which explains its diversification. Pakistani sequences are closely related to the Italian and Indian sequences in the tree.

### Phylogenetic Analysis of HCV Genotype 3b

**Fig 6:**
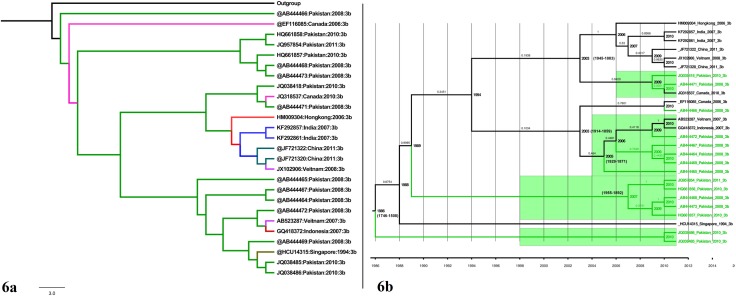
(a) Maximum likelihood phylogeny of NS5B 339bp long fragment gene reconstructed for Pakistani sequence origin of genotype 3b (Highlighted in green) using (GTR+I+G) model. All samples GenBank accession numbers, countries of origin and years of sampling are shown. Individual clades are marked on the right. (b) A time-scaled phylogenetic tree estimated using NS5B 339bp long fragment gene of genotype 3b. Each branch is led by an isolate and is denoted by the following format: GenBank Accession number: Country: Date: Subtype. The branch lengths represent the evolutionary time that is measured by the grids corresponding to the timescale (years) shown at the tree base. The branch labels represent the posterior probability scores. The node labels represent the time of divergence of the respective node. To simplify the tree, the estimated 95% HPD are shown against MRCA and Pakistani clades only.

The phylogenetic history and evolutionary pattern of HCV genotype 3b was studied using NS5B gene (S1 Table). The ML and dated phylogeny of genotype 3b was quiet similar to that of genotype 3a. The trees depicted a polyphyletic origin of the virus though, recurrent clustering of Pakistani sequences together confirmed the frequent inter country dispersal of the virus as seen in Fig 6A. The Pakistani isolates were also seen to group together with Singapore, Vietnam, Indonesia and Canada. The Bayesian phylogeny dated back to 1986 (95% HPD 1746–1508). The divergence date of individual clades is shown in Fig 6B. All clades were well supported statistically.

## Discussion

The current study was carried out to reconstruct the time scaled phylogenies to infer the origin and divergence of HCV genotypes in Pakistan. We applied the ML and Bayesian coalescent based analysis to provide a clear depiction of the problem. We performed analysis of genotype 1a, 1b, 3a and 3b. Majority of infections caused by HCV in the world involves only a few HCV subtypes namely 1a, 1b, 2b, 3a and 4a [31]. These subtypes have disseminated all over the world during the 20^th^ century mainly because of unsafe blood transfusions, injections and intravenous drug abuse [26, 32]. Individual gene analysis using NS5B regions revealed that genotype 3a is the most diverse genotype in Pakistan and thus has been present in the local population for an extensive period of time [33].

For majority of the genotypes analyzed, two types of patterns of HCV transmission in Pakistan were observed. The first pattern reflected the ongoing and sustained transmission or the viral lineage within the country that might be due to a shared transmission route. The other pattern observed represented the individual Pakistani isolates of different HCV subtypes to be more closely related to non- Pakistani isolates. This pattern most likely represents the multiple and sporadic migration of HCV strains. The viral introduction in the country may arise via immigration, travel and tourism [34–36] or through network of injecting drug users [37]. Similar patterns were found in others studies of HCV and HIV [38].

The results revealed that HCV genotype 3a is the most diverse genotype in Pakistan. Similarly, when Pakistan HCV genotype 3a complete genome sequences were analyzed with HCV complete genome sequences of genotype 1, 2, 3, 4, 5 and 6 also reveals the diversity in the Pakistani HCV genotype 3a.

The phylogenetic analysis of genotype 1a depicts that the origin of the virus in the country is polyphyletic. The origin of the genotype was found to be in Ghana which confirms the findings of earlier studies [39]. The plausible reason for the spread of the genotype within the country is via unsafe medical practices. Another study concluded that genotype 1a infected patients got their infection during surgeries. Genotype 1a is most prevalent in Iran, England, Australia and Scotland [40, 41].

Pakistani sequences were found to be closely related to Iran, Italy and Switzerland. A large number of Pakistani population reside in Italy and Switzerland. Whereas, people of specific religious sect cross the Iranian border in large number legally and illegally every year. Our results also indicated that HCV genotype dispersed from the developed countries to under developed countries including Pakistan. The worldwide spread of the virus was initiated and sustained during and after World War II. However, it may also be due to the wide spread of IDU in the 1930s [42] in the western countries and is relatively new to the Asian countries such as the earliest date of divergence for 1a in Pakistan was found to be 2001. These findings coincides with [39] conclusions.

The HCV genotype 1b phylogeny of NS5B region also shows the polyphyletic origin of the virus in Pakistan. The Pakistani isolates are more closely related to Russia, Egypt and New Zealand. Genotype 1b is predominant in Russia [43, 44]. Till date there are thousands of illegal Russian nationals living in Pakistan. They frequently cross the Pakistani border illegally without proper medical screenings. Genotype 1b is not associated with IDU rather its transmission route is more frequently associated to be iatrogenic one. 75% of the patients infected with genotype 1b got infected during surgeries. This may also indicate the insignificant spread of the genotype within the country as now, more safe medical practice is carried out throughout Pakistan. The earliest divergence date for Pakistan was found to be 1925 while the other being as recent as 2010. The origin of 1b was found in West Africa which coincides with the earlier studies [6, 45].

Genotype 3 is the dominant genotype in Pakistan after genotype 1 [46, 47]. It is reported in many studies that genotype 3a has originated in the Indian subcontinent about 300 years ago [48, 49]. Our study also agrees with previous results. The ML and Bayesian phylogenies were well resolved and highly diverse. The high genetic variability as seen in the Pakistani isolates genotype 3a sequences, confirms that 3a has been present in country since long. Previous report [48] suggest that genotype 3 has most probable origin in the northern part of India. Since, India and Pakistan was Indian subcontinent for decades and still share a common boundary due to which they have a large common human ancestry. The individual phylogenetic trees of NS5B region shows that Pakistani sequences tends to cluster together forming a distinct Pakistan specific cluster of genotype 3a. The Bayesian molecular clock analysis provided an estimate of the data of most recent common ancestor (MRCA) of genotype 3a, which is 1992. It was estimated that subtype 3a has a common ancestor dated back to 1992 [95% HPD: 1897, 1273]. The estimated common ancestor of Pakistan HCV genome subtype 3a dated to 2006 [95% HPD: 1988, 1899].

HCV sequences from Pakistan were found to be closely related to Indian sequences in addition to Portugal, Thailand, Vietnam, India, Argentina, UK, Brazil, Russia, Afghanistan and Mexico, Uzbekistan and Switzerland. The earliest divergence date of HCV genotype 3a in Pakistan was found to be 1933. Indian subcontinent has a history of British rule between 1858 and 1947. It is speculated that HCV genotype 3a has disseminated to United Kingdom from the Indian subcontinent during and after the British rule. The time also overlaps with the era of unsafe blood transfusions and rapid industrializations. The spread may also be due to the mass migration of people during World War II. Rehman et al., [33] reported that HCV genotype population increased in Pakistan from 19th century to 1970 by thousand folds. Genotype 3a is very common among IDUs. Moreover, in Pakistan correlation of thalassemia and HCV with genotype 3a has been observed in patients who received blood transfusion several times in their life time. In a recent study by [9, 50] HCV 3a was revealed as the most common genotype in Pakistan. It is also indicated that the epidemic spread of HCV genotype 3a occurred first in Pakistan than in other countries around 1920s and 1950s similar to our results. This spread is also considered as a reason for increased incidence of hepatocellular carcinoma.

Thus, it is proposed that HCV genotype 3a has spread worldwide during 1960s due to widespread use of heroin [16, 51]. It is common practice among Pakistani IDUs to use and re-use needles and syringes with other IDUs. Genotype 3a is less commonly observed in people undergone surgeries or other medical or dental procedures.

HCV genotype 3b is the next most common genotype in Pakistani population. The E1 and NS5B gene analysis to trace the origin of the virus in Pakistan reveals close relationship of Pakistani and Indian sequences. Pakistani sequences were also found to be closely related to other countries including Singapore, Vietnam and Indonesia. The analysis shows dispersal of genotype 3b from Pakistan to China in early 19th century. Pakistan and China have always been on friendly terms and crossing of borders for tourism, study and business is very common. The close clustering of Pakistani sequences in the resulting phylogenies depicts the presence of genotype 3b in Pakistan since a long time. The proportion of genotype 3b in Pakistan has gradually decreased. This can be due to the 100% sustained virological response (SVR) rates recorded among patients of 3a and 3b genotypes [52]. The most common mode of genotype 3b transmission is the same as of genotype 3a i.e. via use and re use of contaminated syringes in IDUs. It has been reported that 60% of the patients infected with genotype 3b were drug addicts using intravenous drug usage.

The growing field of computational phylogenetics is very useful in molecular evolutionary studies. By inferring spatial and temporal processes from viral gene sequences, new insights could be obtained of the epidemiology and spatial diffusion of viruses.

## Conclusions

The goal of this project was to trace and study the origin, the genetic diversity and molecular evolution of HCV population in Pakistan. Our study concludes the origin of HCV of genotype 1a, 1b, 1c, 3a and 3b is polyphyletic. The origin of genotype 3a is Indo-Pak and is highly divergent. The rapid evolution and enormous sequence diversity found in HCV sequences are a cause of a significant hurdle in the development of effective vaccines and novel therapeutic interventions. We tried to gain an insight of the evolutionary aspects of HCV genotypes available in Pakistan. The origin of genotype 3a is Sub-continent Asia which includes Indo-Pak and is highly divergent.

Phylogenetics is very beneficial in molecular evolutionary studies. By applying the spatial and temporal processes from viral genome sequences, new perceptions can be obtained of the epidemiology and spatial diffusion of viruses. The medical fraternity in Pakistan needs to carry on the research in order to provide improved understanding of the clinical trajectory of the disease. Research should also be done to sequence more HCV genomes in Pakistan to compare the nucleotide variation among other countries and target those areas in the genome that are essential for the viral replication like NS3-4A inhibitors and NS5B polymerase inhibitors. Novel therapies should be developed that can be targeted to high-risk populations.

## Supporting Information

S1 Table Table enlists all sequences used to perform the study. (XLSX)

## References

[1] Mohd Hanafiah K, Groeger J, Flaxman AD, Wiersma ST. Global epidemiology of hepatitis C virus infection: new estimates of age-specific antibody to HCV seroprevalence. Hepatology. 2013;57(4):1333–42. Epub 2012/11/23. 10.1002/hep.26141 . 23172780

[2] Raja NS, Janjua KA. Epidemiology of hepatitis C virus infection in Pakistan. Journal of microbiology, immunology, and infection = Wei mian yu gan ran za zhi. 2008;41(1):4–8. 18327420

[3] Neumann AU, Lam NP, Dahari H, Gretch DR, Wiley TE, Layden TJ, et al Hepatitis C viral dynamics in vivo and the antiviral efficacy of interferon-alpha therapy. Science. 1998;282(5386):103–7. Epub 1998/10/02. 10.1126/science.282.5386.103 . 9756471

[4] Domingo E, Escarmis C, Sevilla N, Moya A, Elena SF, Quer J, et al Basic concepts in RNA virus evolution. FASEB journal: official publication of the Federation of American Societies for Experimental Biology. 1996;10(8):859–64. Epub 1996/06/01. . 8666162 10.1096/fasebj.10.8.8666162

[5] Drake JW, Charlesworth B, Charlesworth D, Crow JF. Rates of spontaneous mutation. Genetics. 1998;148(4):1667–86. Epub 1998/04/30. 9560386 10.1093/genetics/148.4.1667 PMC1460098

[6] Simmonds P, Bukh J, Combet C, Deleage G, Enomoto N, Feinstone S, et al Consensus proposals for a unified system of nomenclature of hepatitis C virus genotypes. Hepatology. 2005;42(4):962–73. Epub 2005/09/09. 10.1002/hep.20819 . 16149085

[7] Simmonds P, Holmes EC, Cha TA, Chan SW, McOmish F, Irvine B, et al Classification of hepatitis C virus into six major genotypes and a series of subtypes by phylogenetic analysis of the NS-5 region. The Journal of general virology. 1993;74 (Pt 11):2391–9. Epub 1993/11/01. 10.1099/0022-1317-74-11-2391 . 8245854

[8] Smith JE, Alvarez-Dominguez JR, Kline N, Huynh NJ, Geisler S, Hu W, et al Translation of small open reading frames within unannotated RNA transcripts in Saccharomyces cerevisiae. Cell reports. 2014;7(6):1858–66. Epub 2014/06/17. 10.1016/j.celrep.2014.05.023 24931603 PMC4105149

[9] Kuiken C, Simmonds P. Nomenclature and numbering of the hepatitis C virus Hepatitis C: Springer; 2009 p. 33–53. 10.1007/978-1-59745-394-3_4 19009252

[10] Salemi M, Vandamme A-M. Hepatitis C virus evolutionary patterns studied through analysis of full-genome sequences. Journal of molecular evolution. 2002;54(1):62–70. 10.1007/s00239-001-0018-9 11734899

[11] Yang Z, Rannala B. Molecular phylogenetics: principles and practice. Nature reviews Genetics. 2012;13(5):303–14. Epub 2012/03/30. 10.1038/nrg3186 . 22456349

[12] Ahmad W, Ijaz B, Javed FT, Jahan S, Shahid I, Khan FM, et al HCV genotype distribution and possible transmission risks in Lahore, Pakistan. World journal of gastroenterology: WJG. 2010;16(34):4321 10.3748/wjg.v16.i34.4321 20818816 PMC2937113

[13] Bao Y-p, Liu Z-m, Lu L. Review of HIV and HCV infection among drug users in China. Current opinion in psychiatry. 2010;23(3):187–94. 10.1097/YCO.0b013e328338658b 20308901

[14] Sy T, Jamal MM. Epidemiology of hepatitis C virus (HCV) infection. International journal of medical sciences. 2006;3(2):41 10.7150/ijms.3.41 16614741 PMC1415844

[15] Perz JF, Armstrong GL, Farrington LA, Hutin YJ, Bell BP. The contributions of hepatitis B virus and hepatitis C virus infections to cirrhosis and primary liver cancer worldwide. Journal of hepatology. 2006;45(4):529–38. 10.1016/j.jhep.2006.05.013 16879891

[16] Pawlotsky JM, Roudot-Thoraval F, Bastie A, Darthuy F, Remire J, Metreau JM, et al Factors affecting treatment responses to interferon-alpha in chronic hepatitis C. The Journal of infectious diseases. 1996;174(1):1–7. Epub 1996/07/01. 10.1093/infdis/174.1.1 . 8655978

[17] Hnatyszyn HJ. Chronic hepatitis C and genotyping: the clinical significance of determining HCV genotypes. Antivir Ther. 2005;10(1):1–11. Epub 2005/03/09. . 15751759

[18] Mania A, Kemnitz P, Figlerowicz M, Mozer-Lisewska I, Kowala-Piaskowska A, Wozniak A, et al Clinical picture and liver histology of chronic hepatitis C in children. Infectious Diseases in Clinical Practice. 2012;20(2):141–7. 10.1097/ipc.0b013e3182425b13

[19] Simmonds P. Clinical relevance of hepatitis C virus genotypes. Gut. 1997;40(3):291 10.1136/gut.40.3.291 9135513 PMC1027074

[20] Kearse M, Moir R, Wilson A, Stones-Havas S, Cheung M, Sturrock S, et al Geneious Basic: an integrated and extendable desktop software platform for the organization and analysis of sequence data. Bioinformatics. 2012;28(12):1647–9. 10.1093/bioinformatics/bts199 22543367 PMC3371832

[21] Jukes TH, Cantor CR. Evolution of protein molecules. Mammalian protein metabolism. 1969;3(21):132 10.1016/b978-1-4832-3211-9.50009-7

[22] Darriba D, Taboada GL, Doallo R, Posada D. jModelTest 2: more models, new heuristics and parallel computing. Nature methods. 2012;9(8):772–. 10.1038/nmeth.2109 22847109 PMC4594756

[23] Guindon S, Gascuel O. A simple, fast, and accurate algorithm to estimate large phylogenies by maximum likelihood. Systematic Biology. 2003;52(5):696–704. 10.1080/10635150390235520 14530136

[24] Drummond AJ, Suchard MA, Xie D, Rambaut A. Bayesian phylogenetics with BEAUti and the BEAST 1.7. Molecular biology and evolution. 2012;29(8):1969–73. 10.1093/molbev/mss075 22367748 PMC3408070

[25] Drummond AJ, Rambaut A, Shapiro B, Pybus OG. Bayesian coalescent inference of past population dynamics from molecular sequences. Molecular biology and evolution. 2005;22(5):1185–92. 10.1093/molbev/msi103 15703244

[26] Pybus OG, Charleston MA, Gupta S, Rambaut A, Holmes EC, Harvey PH. The epidemic behavior of the hepatitis C virus. Science. 2001;292(5525):2323–5. 10.1126/science.1058321 11423661

[27] Tanaka T, Kato N, Cho M-J, Shimotohno K. A novel sequence found at the 3′-terminus of hepatitis C virus genome. Biochemical and biophysical research communications. 1995;215(2):744–9. 10.1006/bbrc.1995.2526 7488017

[28] Verbeeck J, Maes P, Lemey P, Pybus OG, Wollants E, Song E, et al Investigating the origin and spread of hepatitis C virus genotype 5a. Journal of virology. 2006;80(9):4220–6. 10.1128/JVI.80.9.4220-4226.2006 16611881 PMC1472033

[29] Drummond AJ, Rambaut A. BEAST: Bayesian evolutionary analysis by sampling trees. BMC evolutionary biology. 2007;7(1):214 10.1186/1471-2148-7-214 17996036 PMC2247476

[30] Swofford DL. {PAUP*. Phylogenetic analysis using parsimony (* and other methods). Version 4.}. 2003.

[31] Ciccozzi M, Zehender G, Cento V, Lo Presti A, Teoharov P, Pavlov I, et al Molecular analysis of hepatitis C virus infection in Bulgarian injecting drug users. J Med Virol. 2011;83(9):1565–70. Epub 2011/07/09. 10.1002/jmv.22154 . 21739447

[32] Pybus OG, Cochrane A, Holmes EC, Simmonds P. The hepatitis C virus epidemic among injecting drug users. Infection, Genetics and Evolution. 2005;5(2):131–9. 10.1016/j.meegid.2004.08.001 15639745

[33] ur Rehman I, Vaughan G, Purdy MA, Xia G-, Forbi JC, Rossi LMG, et al Genetic history of hepatitis C virus in Pakistan. Infection, Genetics and Evolution. 2014;27:318–24. 10.1016/j.meegid.2014.08.005 25131452

[34] Morice Y, Roulot D, Grando V, Stirnemann J, Gault E, Jeantils V, et al Phylogenetic analyses confirm the high prevalence of hepatitis C virus (HCV) type 4 in the Seine-Saint-Denis district (France) and indicate seven different HCV-4 subtypes linked to two different epidemiological patterns. The Journal of general virology. 2001;82(Pt 5):1001–12. Epub 2001/04/12. 10.1099/0022-1317-82-5-1001 . 11297675

[35] Bernier L, Willems B, Delage G, Murphy DG. Identification of numerous hepatitis C virus genotypes in Montreal, Canada. J Clin Microbiol. 1996;34(11):2815–8. Epub 1996/11/01. 8897188 10.1128/jcm.34.11.2815-2818.1996 PMC229409

[36] Stuyver L, Wyseur A, van Arnhem W, Lunel F, Laurent-Puig P, Pawlotsky J-M, et al Hepatitis C virus genotyping by means of 5′-UR/core line probe assays and molecular analysis of untypeable samples. Virus research. 1995;38(2):137–57. 10.1016/0168-1702(95)00052-r 8578855

[37] Cochrane A, Searle B, Hardie A, Robertson R, Delahooke T, Cameron S, et al A genetic analysis of hepatitis C virus transmission between injection drug users. Journal of Infectious Diseases. 2002;186(9):1212–21. 10.1086/344314 12402190

[38] Nakano T, Lu L, He Y, Fu Y, Robertson BH, Pybus OG. Population genetic history of hepatitis C virus 1b infection in China. Journal of General Virology. 2006;87(1):73–82. 10.1099/vir.0.81360-0 16361419

[39] Magiorkinis G, Magiorkinis E, Paraskevis D, Ho SY, Shapiro B, Pybus OG, et al The global spread of hepatitis C virus 1a and 1b: a phylodynamic and phylogeographic analysis. PLoS medicine. 2009;6(12):e1000198 10.1371/journal.pmed.1000198 20041120 PMC2795363

[40] McCaw R, Moaven L, Locarnini SA, Bowden DS. Hepatitis C virus genotypes in Australia. J Viral Hepat. 1997;4(5):351–7. Epub 1997/10/06. 10.1046/j.1365-2893.1997.00060.x . 9310934

[41] Mellor J, Holmes EC, Jarvis LM, Yap PL, Simmonds P. Investigation of the pattern of hepatitis C virus sequence diversity in different geographical regions: implications for virus classification. The International HCV Collaborative Study Group. The Journal of general virology. 1995;76 (Pt 10):2493–507. Epub 1995/10/01. 10.1099/0022-1317-76-10-2493 . 7595353

[42] Stimson GV. The global diffusion of injecting drug use: implications for human immunodeficiency virus infection. Bulletin on narcotics. 1993;45(1):3–17. Epub 1993/01/01. . 8305905

[43] Ray SC, Arthur RR, Carella A, Bukh J, Thomas DL. Genetic epidemiology of hepatitis C virus throughout Egypt. Journal of Infectious Diseases. 2000;182(3):698–707. 10.1086/315786 10950762

[44] Ramia S, Eid-Fares J. Distribution of hepatitis C virus genotypes in the Middle East. International journal of infectious diseases: IJID: official publication of the International Society for Infectious Diseases. 2006;10(4):272–7. Epub 2006/03/28. 10.1016/j.ijid.2005.07.008 . 16564719

[45] Simmonds P. Genetic diversity and evolution of hepatitis C virus–15 years on. Journal of General Virology. 2004;85(11):3173–88. 10.1099/vir.0.80401-0 15483230

[46] Attaullah S, Khan S, Ali I. Hepatitis C virus genotypes in Pakistan: a systemic review. Virol J. 2011;8:433 Epub 2011/09/10. 10.1186/1743-422X-8-433 21902822 PMC3178528

[47] Afridi SQ, Ali MM, Awan F, Zahid MN, Afridi IQ, Yaqub T. Molecular epidemiology and viral load of HCV in different regions of Punjab, Pakistan. Virol J. 2014;11:24 Epub 2014/02/12. 10.1186/1743-422X-11-24 24512668 PMC3925129

[48] Zehender G, Sorrentino C, Lai A, Ebranati E, Gabanelli E, Presti AL, et al Reconstruction of the evolutionary dynamics of hepatitis C virus subtypes in Montenegro and the Balkan region. Infection, Genetics and Evolution. 2013;17:223–30. 10.1016/j.meegid.2013.04.003 23603418

[49] Akkarathamrongsin S, Hacharoen P, Tangkijvanich P, Theamboonlers A, Tanaka Y, Mizokami M, et al Molecular epidemiology and genetic history of hepatitis C virus subtype 3a infection in Thailand. Intervirology. 2013;56(5):284–94. Epub 2013/07/11. 10.1159/000351621 . 23838334

[50] Khan A, Tanaka Y, Azam Z, Abbas Z, Kurbanov F, Saleem U, et al Epidemic spread of hepatitis C virus genotype 3a and relation to high incidence of hepatocellular carcinoma in Pakistan. Journal of medical virology. 2009;81(7):1189–97. Epub 2009/05/29. 10.1002/jmv.21466 . 19475617

[51] Pawlotsky JM. [Methods of study of the hepatitis C virus genome. Diagnostic tools in human pathology]. Veterinary research. 1995;26(1):3–10. Epub 1995/01/01. . 7711774

[52] Idrees M, Riazuddin S. Frequency distribution of hepatitis C virus genotypes in different geographical regions of Pakistan and their possible routes of transmission. BMC Infectious Diseases. 2008;8(1):69 10.1186/1471-2334-8-69 18498666 PMC2409346

